# Segmental origins of the *Drosophila* eye-antennal disc: fission not fusion

**DOI:** 10.1093/genetics/iyac168

**Published:** 2022-11-12

**Authors:** Gary Struhl

**Affiliations:** Department of Genetics and Development, Columbia University, New York, NY 10027, USA

The paired (left/right) eye-antennal discs of the *Drosophila* larva give rise to most of the adult fly head and have been an experimental paradigm for diverse biological phenomena such as appendage development, regeneration, cell patterning, neurogenesis, and sensory perception (visual, olfactory, and tactile). In a recent Flybook chapter “The early history of the eye-antennal disc of *Drosophila melanogaster*,” Weasner and Kumar (2022) tell the story of our growing understanding of this remarkable model system and how it may be advanced using the increasingly powerful genetic and molecular technologies available. Here, I present an argument that in one particular respect, the history has obscured rather than illuminated a fascinating possibility that is ripe for further exploration—namely the capacity of an imaginal disc derived from a single embryonic segment to fission into multiple segments during larval life.

**Fig. 1.**
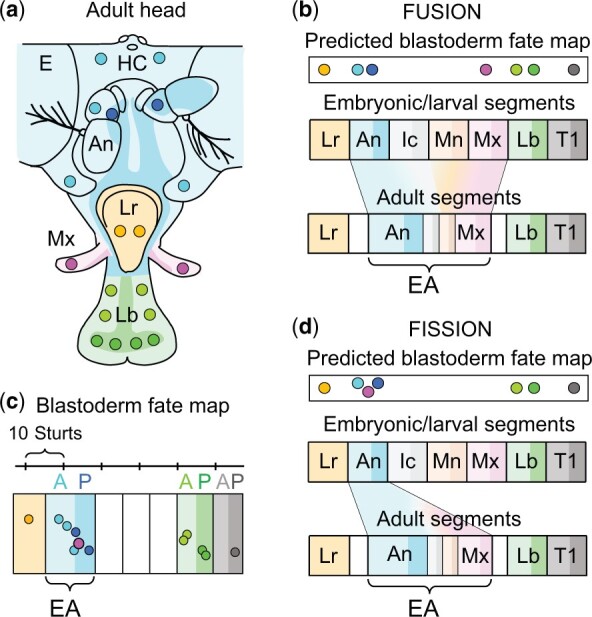
Discriminating between fusion and fission models for the segmental origins of the Drosophila eye-antennal disc. a) Composition of the adult Drosophila head. The adult head is formed by 3 imaginal discs. The labial disc forms the labial palps (Lb, green), the mouth parts for ingesting food, and corresponds to the most posterior of the 3 gnathal segments. The clypeo-labral disc forms the clypeo-labrum (Lr, orange), the pharynx through which food is pumped to the gut, and corresponds to the most anterior of the 3 cephalic segments. The eye-antennal disc forms the rest of the head, which includes the eye (E), antenna (An), and head capsule (HC) (all turquoise), possible intercalary and mandibular structures that contribute to the HC (not indicated), and the maxilla (Mx, purple), a sensory appendage. The labial palps, eye-antenna complex, and maxilla are themselves subdivided into anterior (A) and posterior (P) compartments (shown here and in the remaining panels as lightly and more darkly colored territories; the antenna on the right side is flipped up to help visualize the P compartment; the P compartment of the labial palps contributes to the back side of the structure; the A/P compartmentalization of the clypeo-labrum has not been analyzed). Landmark bristles used for the gynandromorph analysis presented in (c) are indicated as more darkly colored circles. b, d) Fusion and fission models for the segmental origins of the eye-antennal disc. In both models, the labral (Lr), antennal (An), intercalary (Ic), mandibular (Md), maxillary (Mx), labial (Lb), and first thoracic (T1) segments of the embryo derive from a linear series of blastoderm cell populations and develop into the corresponding segmental structures of the larva. In the fusion model (b), a small subset of cells in each of the An, Ic, Md, and Mx segments segregate from the remaining cells during embryogenesis and coalesce into a single, nascent eye-antennal disc that will form the An, Ic, Md, and Mx structures in the adult. In the fission model (d), only cells in the embryonic An segment segregate from the remaining cells to form the nascent eye-antennal disc, which then undergoes a process of *de novo* segmentation into An, Ic, Md, and Mx primordia during larval life. As diagramed, the fusion and fission models make markedly different predictions about the position of the blastoderm progenitor cells of the adult maxilla (purple). In the fusion model the blastoderm progenitors of this structure should map just in front of the labial (green) progenitors (b), but in the fission model they should map with the antennal progenitors (turquoise) and just behind the clypeo-labral (orange) progenitors (d). c) The gynandromorph fate map of blastoderm progenitor cells of landmark bristles in the adult clypeo-labrum, antenna, HC, maxilla, labial palps, and first thoracic segment, color coded as in the remaining panels; from Struhl (1981). The results corroborate the prediction of the fission model, but are not compatible with the prediction of the fusion model.

The adult fly is constructed by imaginal discs that arise as small clusters of embryonic cells that segregrate from surrounding cells destined to form the larva. Once established, the nascent discs proliferate as epithelial sacs during larval life and differentiate the adult structures during metamorphosis. Each imaginal disc typically makes either a dorsal or ventral appendage of a single adult segment, as well as the associated portion of the body wall (Gehring and Nöthiger 1973). However, this is not the case for the eye-antennal disc (Ferris 1950; Gehring 1966; Haynie and Bryant 1986). As depicted in turquoise in Fig. 1a, the eye-antennal disc (EA) is unusual in that it forms both dorsal and ventral organs (the eye, E, and antenna, An), most of the body wall comprising the head capsule (HC), and an additional sensory appendage, the maxilla (Mx, purple). These structures are posited to represent the adult derivatives of 4 embryonic head segments (antennal, An; intercalary, Ic; mandibular, Md, and maxillary, Mx; Jürgens and Hartenstein 1993). In contrast, the remaining 2 adult head structures, the labial palps (Lb, green) and clypeo-labrum (Lr, orange), are each formed by imaginal discs that derive from single embryonic segments (labial and labral, respectively). The composite nature of the eye-antennal disc thus poses the question of whether this disc arises by the fusion of embryonic cell clusters derived from multiple head segments (Fig. 1B), or whether it begins life as a cluster from a single embryonic segment that undergoes fission into multiple segments during subsequent development (Fig. 1D).

Weasner and Kumar present the generally accepted view that the eye-antennal disc arises by fusion. This view is based predominantly on an extrapolation of pioneering HRP injection and laser ablation experiments performed in the 1980s that identified a series of blastoderm cell populations that give rise to well defined head structures of the embryo and first instar larva (Technau and Campos-Ortega 1985; Jürgens *et al.* 1986; Cohen and Jürgens 1991). Using posterior (P) compartment expression of the transcription factor Engrailed (demarcating putative segmental boundaries), as well as HOX selector gene expression (defining segment type), subsequent studies consolidated the assignment of these blastoderm primordia to the well accepted 3 cephalic (Lr, An, Ic) and 3 gnathal (Md, Mx, Lb) segments comprising the basic insect head (reviewed in detail in Jürgens and Hartenstein 1993). Engrailed and HOX gene expression patterns were then used to map the corresponding An, Ic, Md, and Mx domains in the eye-antennal disc and the results presented as evidence that this disc arises during embryogenesis by the coalescence of nascent imaginal cells that segregate from each of these 4 segments (Jürgens and Hartenstein 1993; Younossi-Hartenstein *et al.* 1993). However, this conclusion depends on the presumption that the An, Ic, Md, and Mx domains identified by Engrailed/HOX gene expression in the eye-antennal disc descend from the corresponding An, Ic, Md, and Mx primordia as defined by Engrailed/HOX gene expression in the embryo—a presumption that was not tested in these studies.

Despite its widespread adoption beginning in the 1990s, the fusion model is challenged by earlier gynandromorph fate mapping experiments that counter this presumption and strongly support the fission model (Struhl 1981). Although discounted in discussions of the HRP injection and laser ablation findings (Technau and Campos-Ortega 1985; Jürgens *et al.* 1986; Younossi-Hartenstein *et al.* 1993; Jürgens and Hartenstein 1993), and subsequently omitted from consideration, the gynandromorph data argue instead that the nascent eye-antennal disc derives solely from the embryonic antennal segment and then segregates into multiple segments later in development (Struhl 1981)—a possibility independently supported by other early evidence (Morata and Lawrence 1978, 1979; Haynie and Bryant 1986).

The key challenge to the fusion model rests on the unique power of gynandromorph fate mapping to provide incisive information about the relative positions of the blastoderm progenitor cells of adult landmark structures (Garcia-Bellido and Merriam 1969; Hotta and Benzer 1972). This technology depends on a peculiar genetic anomaly in *Drosophila*: an unstable “ring” X chromosome that is frequently lost by nondisjunction during the first nuclear division following fertilization, generating one daughter nucleus that is male (X/O), and the other female (X/X^ring^). Importantly, the orientation of the cleavage plane of this division is random and the descendant male and female nuclei do not intermix during the subsequent 13 syncytial divisions leading up to the blastoderm stage. As a consequence, the initial cleavage plane is preserved as the proliferating nuclei migrate outwards to form a mono-layer at the egg cortex, after which they are incorporated into cells. By introducing the appropriate recessive mutations on the remaining X chromosome, adult structures such as specific landmark bristles can be scored as descending from either the initial male or female nucleus by changes in cuticle color or morphology.

Accordingly, the probability that the progenitor cells of any 2 landmark structures of the adult will be separated by the cleavage plane at the blastoderm stage depends on their distance from each other: the closer they are, the less likely they will be separated. Hence, these frequencies provide information about the proximity and relative position of the progenitor cells at this stage, and are expressed as percentages termed “sturts” in honor of Alfred Sturtevant, who pioneered the technique (Sturtevant 1929; Garcia-Bellido and Merriam 1969; Hotta and Benzer 1972). Given the segregation of most segments into A (Engrailed OFF) and P (Engrailed ON) compartments at or soon after the blastoderm stage (Steiner 1976; Struhl 1977; Lawrence and Morata 1977), it is possible to determine the minimum sturt distance between landmark structures that derive from adjacent progenitor cells in different compartments within the same segment (∼6–8 sturts), as well as between those that derive from progenitor cells in adjacent segments (∼7–10 sturts). It is also possible to determine the minimum distances between progenitors in 2 segments separated by 1, 2, or 3 intervening segments (∼16, 21, and 25 sturts, respectively).

Of the 4 segmental primordia comprising the mature eye-antennal disc, only those forming the antenna and maxilla differentiate adult landmark bristles that can be mapped by the gynandromorph technique. Despite this limitation, the fusion and fission hypotheses make distinct and mutually exclusive predictions about where the blastoderm progenitors of the antennal and maxillary landmark bristles map relative to each other as well as to the blastoderm progenitors of the labral and labial segments (Figs. 1, b and d; note especially the different predicted positions of the progenitors of the adult maxilla, purple). As shown in Fig. 1c, the results of gynandromorph fate mapping are unequivocal and discriminate between the 2 models. In accordance with the fission model as well as prior mapping data (Garcia-Bellido and Merriam 1969; Hotta and Benzer 1972), the adult maxilla maps to the center of the pool of blastoderm progenitor cells that give rise to the adult antenna (Struhl 1981; corroborated by Haynie and Bryant 1986). Furthermore, the 1981 study showed that this progenitor pool maps (1) just posterior to progenitors of the clypeo-labral disc and (2) far anterior to the progenitors of the labial disc, at a distance corresponding to a gap of 3 intervening segments (∼25 sturts as determined by fate mapping abdominal segments using the same collection of gynandromorphs). In contrast, these results are not compatible with the fusion model in which the adult maxilla is presumed to derive from blastoderm progenitors of the embryonic maxillary segment positioned 2 segments posterior to the progenitors of the antennal segment (a predicted distance of ∼21 sturts) and immediately anterior to the progenitors of the labial segment.

Three main arguments have been made, previously, for discounting the gynandromorph evidence for a fission mechanism. However, these do not, in my opinion, hold up to scrutiny, as described below.

The first is that gynandromorph fate mapping is inherently unreliable as witnessed by different estimates of the number of blastoderm progenitor cells for specific imaginal discs obtained by this method in earlier studies (Younossi-Hartenstein *et al.* 1993). However, the divergent estimates that form the basis of this criticism were not obtained by measuring the sturt distances between the blastoderm progenitor cells of adult landmark bristles. Instead, they were obtained by a different kind of measurement: the frequency of mosaicism observed for the adult derivatives of entire discs. In this case, the adult derivatives of a single imaginal disc are scored as mosaic if any of the chosen landmark bristles are XO whilst the remainder are XX: the larger the pool of blastoderm progenitor cells, the more likely it will be split by the gynandromorph cleavage plane. However, this measure depends critically on whether the chosen adult landmark structures represent the descendants of most or all of the blastoderm progenitors of a given imaginal disc, or only a smaller subset. As discussed at the time, the observed differences in the estimates of progenitor cell numbers based on the frequency of mosaicism likely reflects how well (or not) the chosen landmarks represent the initial pool of blastoderm progenitors (Ripoll 1972; Gehring and Nöthiger 1973; Wieschaus and Gehring 1976a, 1976b). More importantly, this criticism does not apply to the 1981 analysis (Struhl 1981), which measured the sturt distances between specific landmark structures rather than the frequency of mosaicism. As a validation of this approach, these measurements were successful in placing the A compartment founder cells of the labial segment anterior to the P compartment founders, as well as correctly ordering the labral progenitors just in front of the eye-antennal progenitors, and the labial progenitors just in front of the prothoracic (T1) progenitors. It is the unequivocal placement of the progenitors of the adult maxilla within the pool of antennal progenitors and far anterior to the labial progenitors that argues for the fission model and against the fusion model.

The second criticism is the assertion that sturt distances between 2 landmark structures within the same lineage compartment cannot be used to map the relative positions of their progenitors—in contrast to landmark structures in different compartments—because the former can descend from the same founder cells, whereas the latter cannot (Jürgens and Hartenstein 1993; Younossi-Hartenstein *et al.* 1993). This criticism is founded on prior lineage analyses that documented that the antennal and maxillary structures of the adult head come from a shared lineage compartment until mid-way through larval life (Morata and Lawrence 1978, 1979). Accordingly, it has been suggested that once cells from multiple head segments coalesce during embryogenesis to form a nascent eye-antenna disc, most undergo cell death to generate a single lineage population in which the segmental states of the few surviving cells become labile and interchangeable until later, during larval life (Younossi-Hartenstein *et al.* 1993). However, even if one accepts this explanation, the key question remains whether this lineage population derives from a broad swath of blastoderm progenitors spanning the interval between the Lr to the Lb progenitors, or from a discrete progenitor pool corresponding to a single segment. The gynandromorph fate map answers this question by showing that it comes from a single segmental pool, the blastoderm progenitors of the An segment, in accord with the fission model as diagramed in Fig. 1d.

The third criticism is that the fission hypothesis depends on the a priori assumption that if all cells within a single imaginal disc have a shared, group lineage, they must descend from the embryonic primordium of a single segment, in contrast to the fusion hypothesis in which multiple segments are posited to make contributions to a single disc (Jürgens *et al.* 1986; Diederich *et al.* 1991; Jürgens and Hartenstein 1993). However, gynandromorph mapping makes no such assumption. It merely establishes the relative positions of the blastoderm progenitors of well-established Lr, An, Mx, and Lb structures of the adult head. The fission hypothesis provides an explanation for these results that is consonant with the experimental findings, whereas the fusion hypothesis does not.

Although the gynandromorph mapping data is the key evidence arguing in favor of the fission model and against the fusion model, it is important to note that it is supported by 3 additional and independent lines of experimental evidence. First, early lineage studies established that unlike all the other imaginal discs, the eye-antennal disc segregates into A and P developmental compartments—a hallmark of segmentation—surprisingly late, during mid-larval life, rather than at the onset of gastrulation (Morata and Lawrence 1978, 1979), as expected if it fissions into multiple segments during this later stage. This finding is also concordant with the correspondingly late deployment of Engrailed (Brower 1986; Hama *et al.* 1990), which is critical for A/P compartmentalization, as well as the absence of a lineage segregation that delineates the antennal and maxillary primordia as distinct segments prior to the A/P segregation (Morata and Lawrence 1978, 1979; Lebreton *et al.* 2008). Second, the head-specific HOX genes that are expressed in the embryonic progenitors of the larval head segments (notably the Antennapedia-Complex genes *labial*, *Deformed*, and *proboscipedia*) are not expressed in the embryonic cells that are set aside to form the nascent eye-antenna disc (Younossi-Hartenstein *et al.* 1993). Instead, expression of these genes, which specify segmental states in the adult head, is only first observed in the eye-antennal disc around the time the A/P segregation occurs (Diederich *et al.* 1991; Chouinard and Kaufman 1991; Lebreton *et al.* 2008; reviewed in Jürgens and Hartenstein 1993), or in the case of *proboscipedia* even later, during the pupal period (Percival-Smith *et al.* 1997; Benassayag *et al.* 2003; Percival-Smith *et al.* 2017). All 3 of these findings corroborate and extend the gynandromorph fate map data arguing that the eye-antennal disc derives from a single embryonic segment that fissions into multiple segments during larval life.

Why is this seemingly obscure question of the blastoderm fate map of the adult fly head of interest? In my view, it points to an unexpected and fascinating biological phenomenon—the capacity of a single segmental primordium to segregate into multiple segments de novo and to redeploy HOX genes to specify their different states. This phenomenon has been obscured by the predominant fusion dogma. How segmentation might arise, instead, by fission constitutes yet one more intriguing biological mystery posed by the eye-antenna disc. Given the remarkable evolutionary conservation of HOX gene function, solving this mystery may have general implications that go beyond the particular case of the fly head.
